# Cumulative trauma and other determinants of post-traumatic stress disorder, anxiety and depression in medical students following the Great Anatolian earthquake in Turkey

**DOI:** 10.1007/s00127-025-02876-6

**Published:** 2025-03-25

**Authors:** Fatma Tuygar-Okutucu, Hacer Akgul Ceyhun

**Affiliations:** Department of Psychiatry, Ataturk University Medical Faculty, Erzurum, 25240 Turkey

**Keywords:** Cumulative trauma, Post-traumatic stress disorder, Anxiety, Depression

## Abstract

**Background:**

Following the 2023 Turkey earthquake, university students in the earthquake district were transferred to other universities and our university was one of those. In addition, the families of many of our students were living in the earthquake district, and they were with their families during the earthquake due to the semester. We created a trauma psychiatry policlinic to serve medical students and others affected by the disaster. To identify students affected and to provide support, we conducted a cross-sectional study on medical students two months after the earthquake. We aimed to evaluate the prevalence rate and cumulative trauma and other determinants of post-traumatic stress disorder (PTSD), anxiety, and depression.

**Methods:**

A cluster sampling procedure was used. In addition to generating socio-demographic and earthquake related dataform, PTSD checklist-5, Cumulative Stress and Trauma Scale (CST-S), and Beck Anxiety Inventory, Beck Depression Inventory were administered. All results were evaluated statistically.

**Results:**

A total of 617 medical students participated in the study. PTSD, anxiety, and depression rates were 38.9%, 28.7%, and 21.1% respectively. Gender, previous psychiatric diagnosis, and high scores of earthquake-related features were significant for three. Negative scores of survival, personal identity, collective identity, and family-attachment trauma sub-types of CST-S were associated with all three diagnoses.

**Conclusions:**

Negative scores of the survival, personal identity, collective identity, and family attachment trauma subtypes of the CST-S are associated with all three diagnoses. However, these results require to be supported by longitudinal studies.

## Introduction

Two earthquakes with magnitudes of 7.7 and 7.6 on the Richter scale that occurred one after another in Kahramanmaras, Turkey on February 6 in 2023 directly affected 11 provinces and more than 13 million citizens. Hundreds of aftershocks continued for months. More than 50.000 people died [1].

A significant rate of students were either residing or receiving education in the earthquake district. The Higher Education Institution reported that students were among the most affected groups. Universities in earthquake district were matched with 8 other universities to be transferred in order to make academic and administrative assignments [2]. In this context, our university (located close to earthquake district) was matched with an earthquake district university and a total of 900 guest students were transferred to our Medical Faculty.

In our hospital, we created a trauma psychiatry policlinic that served medical faculty students and others affected by the disaster. In order to identify students affected by the disaster and to provide support, we decided to screen all, specifically for the most common post-disaster diagnoses, post-traumatic stress disorder, anxiety, and depression.

Post-Traumatic Stress Disorder (PTSD) is a common psychiatric problem that occurs as a result of an event/events that are so painful/stressful that they pose an exceptional threat to a person’s life [3]. The individual may develop symptoms, such as intrusive thoughts of different aspects of the traumatic event, feelings of helplessness, intense fear, frightening dreams, or avoiding the source of the trauma. These symptoms seriously impair the functions of daily life. The determinants/predictors of PTSD, depression, and anxiety following exposure to a disaster are numerous [4, 5].

Variables such as age, gender, pre-disaster and post-disaster traumatic events, persistent violence, and peritraumatic distress are main predictors of PTSD, depressive, and anxiety symptoms in earthquake survivors [6, 7].About 50% of PTSD symptoms last more than 3 months and turn into a chronic illness. Chronic PTSD causes social and occupational difficulties, can increase the risk of other mental disorders, even suicide. PTSD often shows comorbidity with depression and anxiety [8].

One of the determinants in formation of PTSD, depression and anxiety is cumulative trauma. Research has provided evidence of heterogeneous effects of potentially traumatic experiences on mental health [9]. Models of sensitization of trauma exposure suggest that prior trauma experiences may increase susceptibility to negative consequences following exposure to subsequent potentially traumatic experiences [10].

Kinzie et al. [11] investigated the impact of exposure to a recent potentially traumatic event on previously traumatized refugees with and without pre-existing PTSD. Two months after exposure to additional trauma, they found an increase in post-traumatic stress symptoms in all participants.

To add to the limited literature on the predictors of trauma-related mental disorders among medical school students living in our country, this study examines the impact of the 2023 Great Anatolian earthquake on the prevalence of PTSD, depression, and anxiety, and researches the question as to whether socio-demographic, earthquake exposure-related and clinical factors such as cumulative trauma are associated with the presence of mental disorders. It has been hypothesized that the prevalence of PTSD, depression, and anxiety will increase following the earthquake. It has been also hypothesized that socio-demographic, exposure-related, and cumulative trauma characteristics would predict the presence of PTSD, depression, and anxiety among medical students affected by the earthquake in Turkey. To our knowledge, this is the first study conducted in our country regarding this earthquake. Although there is research on earthquakes and their impact on PTSD, depression, and anxiety, there are not many studies evaluating the relationship between emerging mental disorders and cumulative trauma.

## Methods

### Participants

Universities in the earthquake district were matched with other universities to be transferred, our university (located close to the earthquake district) was one of those and a total of 900 medical students from earthquake district were transferred to our Medical Faculty. In addition, families of many of our students were living in the earthquake district, and they were with families during the earthquake due to semester. We conducted a cross-sectional study, evaluated the prevalence rate of PTSD, depression, anxiety and associations with socio-demographic, earthquake exposure-related, and cumulative trauma characteristics of the participants.

This cross-sectional study was conducted in our medical faculty (located close to earthquake region and accepted guest students) two months after the Great Anatolian Earthquake of Turkey.

The research was approved by the Ethics Committee of Ataturk University Faculty of Medicine (2/86-2023). Collected using a Microsoft Forms questionnaire distributed to students via e-mail followed by a reminder and self-administered. All participants signed informed consent forms.

### Procedure

A cluster sampling procedure was used to generate a representative sample of medical students attending school in grades 1–6. Power analysis was done to determine the sample size. Our sample size was 590 participants, with 80% effect size, 5% alpha error, and 20% beta error with the power analysis determined by the G*power (version 3.1.9.7) program, according to a reference study evaluating the prevalence, comorbidity and predictors of post-traumatic stress disorder, depression, and anxiety in adolescents following an earthquake [3].

Considering the possibility of them not attending, we invited via e-mail a total of 720 students in 6 classes, 120 students from each class. 650 students filled out the questionnaires. We also excluded those with missing data. We analyzed the data of 617 students. 350 students were in preclinical classes and 267 were in clinical classes.

The DATA of 617 students who filled out the questionnaires and scales between April 2023 and June 2023 was analyzed.

**Inclusion criteria;** 18 years or older, still receiving preclinical/clinical training, volunteering to participate.

**Exclusion criteria;** not giving consent for participation and with missing data.

### Measures

An assessment tool was used to generate data. The tool has six sections: demographic information, earthquake related characteristics, screening for PTSD, depression and anxiety with PCL-5, Beck Depression Inventory (BDI) and Beck Anxiety Inventory (BAI) repectively and Cumulative Stressors and Traumas Scale short form (CST-S) for evaluation of cumulative trauma and sub-types.

### Sociodemographic and earhquake related characteristics

A sociodemographic and earhquake-related dataform was developed by the authors to evaluate demographic and earthquake related characteristics. The dataform prepared in accordance with the literature for earthquake-related characteristics was administered as a sub-form of sociodemographic dataform.

### Post-traumatic stress disorder

PTSD was screened using PCL-5. It was revised by Weathers et al. (2013) [12]. according to DSM-5, consists of 20 items. Scoring is between 0 and 4 on the 5-point Likert-type scale, which evaluates level of symptoms in last 1 month. Total score ranges from 0 to 80, and higher scores indicate greater PTSD symptom severity [13]. Items with 2 or more points from each of the questions 1–5, 6–7, 8–14, 15–20 are considered to meet B, C, D, E items criteria in DSM-5 respectively and PTSD diagnosis can be made. Criteria B includes intrusive thoughts, flashbacks, bad dreams/nightmares, Criteria C includes avoidance symptoms, Criteria D includes symptoms of negative thoughts and feelings, Criteria E includes sleep problems, irritability, concentration difficulties, hypervigilance, exaggerated startle response. Validity and reliability study was conducted by Boysan et al. The Cronbach α reliability coefficient of the scale was calculated as 0.96 for the combined scale, while the reliability of the subscales was estimated as 0.89, 0.85, 0.90 and 0.89, respectively [14].

### Depression

Depression level was screened using Beck Depression Inventory (BDI), a self-administered scale which consists of 21 questions and 4-point Likert type items [15]. Each item receives an increasing score between 0 and 3. The scores are as: minimal range = 0–13, mild = 14–19, moderate = 20–28, severe = 29–63. Turkish validity and reliability study was conducted by Hisli et al. (1989) [16]. In the reliability study of the Turkish form of the scale, the internal consistency Cronbach alpha coefficient was found to be 0.86 [16]. In our study, score of 19 and above were considered to have depression.

### Anxiety

Anxiety was assessed using the Beck Anxiety Inventory (BAI) [17]. Turkish validity and reliability were determined by Ulusoy et al. and internal consistency Cronbach alpha coefficient value was found to be 0.93 and reliability was 0.75 [18]. It is a 21-item scale consisting of 4-point Likert type items. Each question is scored between 0 and 3. The cut-off values are normal between 0 and 7 points, mild between 8 and 15 points, moderate between 16 and 25 points, and severe between 26 points and above. In our study, score of 17 and above were considered to have anxiety.

## Cumulative trauma

It was assessed using Cumulative Stressors and Traumas Scale (CST-S). It was developed by Kira et al. [19]. It includes 35 items. Items include different types of trauma including collective identity trauma, family attachment trauma, survival trauma, personal identity trauma. For each item, it consists of three evaluation parameters: the occurrence of event, degree of being affected by the event, and the age at which it first occurred. In the event occurrence parameter, it is evaluated as never = 0, once = 1, twice = 2, three times = 3 and many times = 4. It is evaluated between 1(positive)-7(negative) degree.

It creates the composite score by multiplying the occurrence of the event and degree of being affected. Combined scores are obtained by multiplying the occurrence of event with the positive/negative scores. Positive evaluation can be less symptoms and more predictive of post-traumatic growth [19]. In addition, cumulative trauma can be a stronger predictor of negative evaluations than cumulative trauma [20]. For this reason, positive-negative evaluations were included in the analysis in this study.

The first Turkish adaptation of CST-S was made by Eltan (2022) [21]. It consists of four factor structures: survival traumas (ST), personal-identity traumas (PIT), collective-identity traumas(CIT), and family-attachment traumas (FAT). In the CST-S negative evaluation parameter, the Croncbach Alpha Coefficient was found acceptable as 0.74 for women, 0.75 for men, and 0.74 for all samples. The Croncbach Alpha Coefficient in the positive evaluation parameter was found to be 0.60 for men, 0.35 for women and 0.58 for the whole sample. According to the study sample of the scale, the Croncbach Alpha Coefficient value was found to be 0.823 [21].

### Data analysis

Statistical analyzes were performed with SPSS 25.0. The conformity of variables to normal distribution was examined by histogram graphics and Kolmogorov-Smirnov test. Mean, standard deviation, median, min-max values were used while presenting descriptive analyzes. Categorical variables were compared with Chi-Square Test. The Mann Whitney U Test was used for nonparametric variables. Spearman Correlation Test was used in the analysis of measurement data with each other. Logistic regression analyzes were performed to examine the predictors of PTSD, depression, and anxiety.

## Results

We recruited 617 medical students including 237(37.4%) men and 380(61.6%) women in the study. The mean age was 22.20 ± 2.37. 119(19.3%) participants were students transferred from the university in earthquake region, and 498(80.7%) were from our university. 159(25.8%) were residing in the earthquake region, 458(74.2%) were not.

607(98.4%) were single, 517(83.8%) had a medium income rate, 131(21.2%) were smoking, 70(11.3%) were using alcohol, 73(11.8%) had a systemic disease, 554(89.8%) were living in a city center, 91(14.7%) had a previous psychiatric history, 108(17.5%) had previous psychiatric history of family.

We secondly re-grouped the students who were there during the earthquake and those whose families were there as the earthquake-exposed group. Then evalauted the socio-demographic and clinical variables of the earthquake-exposed group. There were 252 (40.8% of all) earthquake-exposed students including 146(57.9%) women and 106(42.1%) men. The mean age was 22.14 ± 2.84. 159(63.1%) of them were there themselves, 93(36.9%) of them had their families there during the earthquake.

There was no significant difference between the earthquake exposed and non-exposed groups in terms of sociodemographic characteristics.

### Earthquake related characteristics

160(25.9%) students reported being there during the earthquake. 461(74.7%) of total reported being in own hause, 386(62.6%) of them were with their family. 3(0.5%) students reported staying under rubble, 13(2.1%) reported staying under rubble of family and 152(24.6%) reported staying under rubble of their relatives/neighbours. 91(14.7%) sudents had witnessed to rescue, 79(14.9%) to death, 129(20.9%) to injury, 53(9.6%) had participated in search and rescue. 13(2.1%) reported living in a prefabricated settlement for a while. 104 (16.9%) reported financial loss, 196(31.8%) reported damage of home. 53(8.6%) had taken psychosocial support.

There significant differences between the earthquake exposed and non-exposed groups in terms of earthquake-related characteristics.

### Evaluation of cumulative trauma

CST-S scores were evaluated in four subtypes as ST, PIT, CIT, FAT and total scores according to positive and negative evaluation scores.

### Prevalence of PTSD, depression and anxiety

A total of 240(38.9%) participants were evaluated as likely PTSD, 177(28.7%) participants were evaluated as likely anxiety and 130(21.1%) participants as likely depression.

### Socio-demographic and clinical variables for participants with PTSD, depression and anxiety

Participants were divided into three groups as diagnosed with PTSD, depression and anxiety. Groups were compared in terms of socio-demographic and clinical characteristics for diagnosed and undiagnosed participants.

Age was significant only for anxiety group, not depression and PTSD compared to undiagnosed groups. The mean age of PTSD group was 22.23 ± 2.41(*p* =.524), anxiety group was 21.87 ± 2.09(*p* =.010) and depression group was 22.13 ± 2.28(*p* =.382).

The rate of women was higher than men in all three. Suicide history, previous psychiatric history, family psychiatric history, psychiatric complaints and drug use after earthquake were significantly higher in participants with PTSD, anxiety and depression.

While smoking was not significant for anxiety(*p* =.315), higher for depression(*p* =.001) and PTSD(*p* =.033). Alcohol was not significant in any. The economic situation was not significant for PTSD(*p* =.066), but low economy rate was higher for anxiety(*p* =.040) and depression(*p* =.000).

Being residing in earthquake province, and being there during earthquake were higher for all three diagnoses.

Undiagnosed individuals had higher rates of being at home during earthquake, staying at home and communicating after earthquake.

Rate of witnessing to rescue, injury, death, death of a family member, participating in search and rescue efforts, and having a close relative or neighbour under debris, were high for all three groups.

Rate of houses destroyed or with no damage were more common for undiagnosed, while rate of mild-moderate-heavy damaged hauses was higher in three of the diagnosed groups.

Where they were in the earthquake, whether they were alone, whether their house was detached or not, on which floor they were, were not meaningful for any.

Anxiety total scores were higher in PTSD (mean ± SD = 3.52 ± 10.16,*p* =.000) and depression (mean ± SD = 29.86 ± 14.16,*p* =.000) groups. Depression total scores were also higher in PTSD (mean ± SD = 29.35 ± 7.77,*p* =.000) and anxiety (mean ± SD = 29.35 ± 7.77,*p* =.000) groups.

In terms of cumulative trauma scores; Negative scores of CST-S total, ST, PIT, CIT, FAT were significantly higher in all three. There was no significant difference of positive cumulative trauma scores between the groups.

Socio-demographic and clinical variables of earthquake exposed group and participants with PTSD, depression and anxiety are illustrated in Table 1.

**Table 1:** Socio-demographic and clinical variables of earthquake exposed group and PTSD, anxiety and depression groups

	Earthquake exposed group *n*=252, 40.8%	p	Likely PTSD *n*=240, 38.9%	p	Likely Anxiety *n*=177, 28.7%	p	Likely Depression *n*=130, 21.1%	p
**Age**	22.14±2.84	0.594	22.23±2.41	0.524	21.87±2.09	0.010*	22.13±2.28	0.382
**Gender**								
Women Men	146(57.9%) 106(42.1%)	0.121	192(80.3%) 48(19.7%)	0.000***	130(73.4%) 47(26.6%)	0.000***	94(72.3%) 36(27.7%)	0.010*
**Marital status**								
Single Married	249(98.8%) 3(1.2%)	0.482	240(100%) 0(0%)	0.250	177(100%) 0(0%)	0.375	129(99.2%) 1(0.8%)	0.231
**Income**								
Low Medium High	29(11.5%) 210(83.3%) 13(5.2%)	0.003**	33(14.1%) 197(81.7%) 10(4.2%)	0.066	18(10.2%) 151(85.3%) 8(4.5%)	0.040*	19(14.6%) 108(83.1%) 3(2.3%)	0.000***
**Which university**				.				
Host Guest	133(52.8%) 119(47.2%)	. 000***	189(78.8%) 51(21.2%)	000***	115(65%) 62(35%)	0.000***	88(67.7%) 42(32.3%)	0.000***
**Living localization**								
-Village -Town -City	13(5.2%) 16(6.3%) 223(88.5%)	0.360	23(9.9%) 5(1.4%) 212(88.7%)	0.081	15(8.5%) 10(5.6%) 152(85.9%)	0.020*	13(10%) 2(1.5%) 115(88.5%)	0.006*
**Smoking**								
Yes No	55(21.8%) 197(78.8%)	0.764	75(31%) 165(69%)	0.033*	42(23.7%) 135(76.3%)	0.315	41(31.5) 89(68.5)	0.001**
**Alcohol**								
Yes No	28(11.1%) 224(88.8%)	0.879	41(16.9%) 199(83.1%)	0.118	23(13%) 154(87%)	0.416	19(14.6%) 111(85.4%)	0.184
**Systemic disease**								
Yes No	34(13.5%) 218/(86.2%)	0.289	57(23.9%) 183(76.1%)	0.000***	28(15.7%) 149(84.2%)	0.053	25(19.2%) 105(80.8%)	0.002**
**Suicide history** Yes No	10(4%) 242(96%)	0.288	24(9.9%) 216(90.1%)	0.000***	12(6.8%) 165(93.2%)	0.000***	13(10%) 117(90%)	0.000***
**Psychiatric history**								
Yes No	38(15.1%) 214(84.9%)	0.847	84(35.2%) 156(64.8%)	0.000***	48(27.1%) 129(72.9%)	0.000***	42(32.3%) 88(67.7%)	0.000***
**Psychiatric history of family**						.		
Yes No	48(19%) 204(81%)	0.402	84(35.2%) 156(64.8%)	0.000***	43(24.3%) 134(75.7%)	000***	40(30.8%) 90(69.2%)	0.000***
**Increase in psychiatric complaints after earthquake**								
Yes No	64(25.4%) 188(74.6%)	0.000***	128(53.5%) 112(46.5%)	0.000***	65(36.7%) 112(63.3%)	0.000***	48(36.9%) 82(63.1%)	0.000***
**Increase in psychiatric drug after earthquake**								
Yes No	9(3.6%) 243(96.4%)	0.071	31(12.7%) 209(87.3%)	0.000***	11(6.2%) 166(93.8%)	0.000***	9(6.9%) 121(93.1%)	0.000***
**Were they there during the earthquake?**								
Yes No	159(63.1%) 93(36.9%)	0.000***	132(54.9%) 108(45.1%)	0.000***	77(43.5%) 100(56.5%)	0.000***	54(41.5%) 76(58.5%)	0.000***
**Where were during the earthquake?**								
- No information -In own home -In a house other than own -In a cafe or other social setting -Car -Open area	6(2.4%) 176(69.8%) 61(24.2%) 4(1.6%) 4(1.6%) 1(0.4%)	0.277	8(2.8%) 160(67.6%) 60(25.4%) 4(1.4%) 4(1.4%) 4(1.4%)	0.473	4(2.3%) 130(73.4%) 39(22%) 2(0.6%) 1(1.1%) 2(0.6%)	0.277	3(2.3%) 89(68.5%) 30(23.1%) 2(1.5%) 4(3.1%) 2(1.5%)	0.196
**Is the house detached?**								
Yes No	42(16.7%) 210(83.3%)	0.066	35(15.5%) 205(85.5%)	0.628	28(17.8%) 149(84.2%)	0.207	17(13.1%) 113(86.9%)	0.895
**Which floor**								
0-3 4-6 7 and above	108(60,7%) 83(33%) 16(6,3%)	0.201	148(62%) 68(28.2%) 24(9.8%)	0.113	112(63.3%) 55(31.0%) 10(5.7%)	0.081	79(77.4%) 41(43.2%) 10(7.7%)	0.214
**Alone during the earthquake**								
Yes No	62(24.6%) 190(75.4%)	0.174	57(23.9%) 183(76.1%)	0.661	44(24.9%) 133(75.1%)	0.248	32(24.6%) 98(75.4%)	0.426
**Who was with you**								
Unknown Family Neighbour Relatives Unknown persons	43(17.1%) 146(57.9%) 14(5.6%) 11(4.4%) 38(15.1%)	0.004**	41(16.9%) 148(62%) 14(5.6%) 10(4.2%) 27(11.3%)	0.908	33(18.6%) 117(66.1%) 5(2.8%) 5(2.8%) 17(9.6%)	0.672	25(19.2%) 80(61.5%) 4(3.1%) 3(2.3%) 18(13.8%)	0.586
**Staying under rubble**								
Yes No	3(1.2%) 249(98.8%)	0.037*	2(0.8%) 238(99.2%)	0.531	0(0%) 177(100%)	0.239	1(0.8%) 129(99.2%)	0.648
**Staying under rubble of family**								
Yes No	13(5.2%) 239(94.8%)	0.000***	10(4.2%) 230(95.8%)	0.187	4(2.3%) 173(97.7%)	0.726	5(3.8%) 125(96.2%)	0.064
**Staying under rubble of relatives-neighbours**								
Yes No	179(29%) 73(71%)	0.000***	125(52.1%) 115(47.9%)	0.000***	74(41.8%) 103(58.2%)	0.000***	54(41.5%) 76(58.5%)	0.000***
**Witnessing to the rescue**								
Yes No	91(63.6%) 159(36.4%)	0.000***	57(23.2%) 183(76.8%)	0.045	38(21.5) 139(78.5%)	0.001***	28(21.5%) 102(78.5%)	0.004**
**Witnessing to death**								
Yes No	79(13.1%) 171(86.9%)	0.000***	54(22.5%) 186(77.5%)	0.000***	36(20.3%) 141(79.7%)	0.000***	28(21.5%) 102(78.5%)	0.000***
**Witnessing to injury**								
Yes No	129(21.4%) 475(78.6%)	0.000***	135(56.3%) 105(43.7%)	0.000***	60(33.9%) 117(66.1%)	0.000***	45(34.6%) 85(65.4%)	0.000***
**Death of family member**								
Yes No	132(52.2%) 120(47.8%)	0.000***	138(57.7%) 102(42.3%)	0.000***	62(35%) 115(65%)	0.000***	49(37.7%) 81(62.3%)	0.000***
**Communication with others**								
Yes No	132(52.2%) 120(47.8%)	0.000***	138(57.7%) 102(42.3%)	0.001**	88(49.8% 89(50.2%)	0.000***	63(49.5%) 67(51.5%)	0.001**
**Participation in search and rescue**								
Yes No	50(19.8%) 202(80.2%)	0.000***	44(18.3%) 196(81.7%)	0.002**	21(11.9%) 156(88.1%)	0.046*	17(13.15%) 113(86.9%)	0.011*
**Where was after the earthquake?**								
-In own home -At relatives’ house -In the place allocated by the social support institution -In the tent city -Went out of town	110(43.7%) 55(21.8%) 15(6%) 15(6%) 57(22.6%)	0.000***	105(43.7%) 61(25.4%) 20(8.5%) 7(2.8%) 47(19.7%)	0.000***	100(56.5%) 34(19.2%) 10(5.6%) 7(4.0%) 26(14.7%)	0.000***	18(13.9%) 59(45.4%) 21(16.2%) 6(4.6%) 26(20%)	0.000***
**Prefabricated settlement**								
Yes No	21(5.3%) 231(94.7%)	0.000***	14(5.8%) 226(94.2%)	0.035*	6(3.4%) 171(96.6%)	0.263	6(4.6%) 124(95.4%)	0.022*
**Financial loss**								
Yes No	110(42.3%) 242(57.7%)	0.000***	135(56.3%) 105(43.7%)	0.000***	58(32.8%) 119(67.2%)	0.000***	41(31.5%) 89(68.5%)	0.000***
**Damage at home**								
-Destroyed -Heavy damage -Medium damage -Mild damage -No damage	14(5.6%) 30(11.9%) 24(9.5%) 78(31%) 106(42.1%)	0.000***	10(4.2%) 41(16.9%) 27(11.3%) 51(21.1%) 111(46.5%)	0.000***	14(7.9%) 17(9.6%) 13(7.3%) 35(19.8%) 98(55.4%)	0.000***	11(12.5%) 13(6.5%) 14(4.9%) 26(17%) 66(89.1%)	0.000***
**Psychosocial support**								
Yes No	21(5.7%) 231(94.3%)	0.008**	56(14.1%) 194(85.9%)	0.376	13(24.3%) 164(75.7%)	0.000***	12(9.2%) 118(90.8%)	0.000***
BAI total score	20.04±14.74	0.000***	3.52±10.16	0.000***	33.27±9.86	0.000***	29.86±14.16	0.000***
BDI total score	16.41±11.16	0.000***	29.35 ±7.77	0.000***	2186±10.82	0.000***	28.82±8.34	0.000***
PCL-5 B	12.25±7.75	0.000***	14.74±4.07	0.000***	11.88±5.51	0.000***	12.79±5.12	0.000***
PCL-5 C	10.00±5.37	0.000***	5.90±1.70	0.000***	4.66±2.53	0.000***	5.05±2.32	0.000***
PCL-5 D	3.83±2.42	0.000***	19.69±5.35	0.000***	15.27±7.80	0.000***	16.92±7.50	0.000***
PCL-5 E	10.64±7.07	0.000***	17.61±4.16	0.000***	13.18±6.89	0.000***	14.73±6.28	0.000***
PCL-5 total score	36.48±21.89	0.000***	59.39±11.68	0.000***	48.67±16.70	0.000***	52.01±1.50	0.000***
STP	1.15±3.96	0.388	0.89±1.80	0.079	0.84±2.39	0.299	0.89±1.75	0.505
STN	12.71±12.10	0.000***	16.86±15.72	0.000***	13.00±13.51	0.000***	14.11±13.41	0.000***
PITP	1.12±2.51	0.711	1.00±1.96	0.862	1.10±3.13	0.775	0.90±1.86	408
PITN	11.69±16.90	0.016*	22.74±20.07	0.000***	16.71±19.57	0.000***	21.75±21.40	0.000***
CITP	0.85±3.07	0.042*	12.66±16.90	0.118	0.57±2.90	0.919	0.42±1.45	0.340
CITN	7.34±12.37	0.000***	0.48±1.56	0.000***	9.08±13.72	0.000***	11.08±14.78	0.000***
FATP	0.26±1.17	0.618	0.35±1.00	0.380	0.29±1.07	0.315	0.30±1.07	0.395
FATN	8.26±8.70	0.000***	10.25±10.25	0.000***	8.06±8.75	0.000***	8.22±9.16	0.001**
CTS-S P	4.32±9.17	0.190	51.17±34.42	0.528	3.36±11.12	0.947	2.28±5.23	0.483
CTS-S N	34.85±31.59	0.000***	3.29±4.88	0.000***	40.69±32.57	0.000***	45,29±29,53	0.000***

Earthquake victims are compared to non-earthquake victims, PTSD group is compared to non-PTSD group, Anxiety group is compared to non- Anxiety group and Depression Group is compared to non-Depression group

BAI: Beck Anxiety Inventory BDI: Beck Depression Inventory PCL-5: Posttravmatik Stress Disorder Checklist for DSM-5 STP: Survival Traumas positive STN: Survival Traumas negative PITP: Personal Identity Traumas positive PITN: Personal Identity Traumas negative CITP: Collective Identity Traumas positive CITN: Collective Identity Traumas negative FATP: Family-Attachment Traumas positive FATN: Family-Attachment Traumas negative CTS-S P: Cumulative trauma positive CTS-S N: Cumulative trauma positive

*p <.05; **p <.01; ***p <.001

### Correlations of PTSD, anxiety and depression scores with CST-S and other clinical characteristics

Age and school grade were negatively correlated with PCL-5 B (*r*=-.181,*p* =.000); C (*r*=-.154,*p* =.000); D (*r*=-.179,*p* =.000); E (*r*=-.173,*p* =.000), PCL-5 total scores (*r*=-.137,*p* =.001) and BAI (*r* =.562,*p* =.000) scores.

BDI was not associated with age (*r*=-.023, *p* =.585), but negatively correlated with school grade (*r*=-.091,*p* =.028).

PCL-5 total and B, C, D, E subscores were positively correlated with STN, PITN, CITN, FATN, and CST-S total. A moderately significant positive correlation was detected only in D score with STP.

While BAI and BDI total scores were positively correlated with STN, PITN, CITN, FATN, not correlated with positive scores.

Correlations of PTSD, anxiety and depression scores with CST-S and other clinical characteristics of all participants are illustrated in Table 2.

**Table 2:** Correlations of PTSD, anxiety and depression scores with CST-S scores and other clinical characteristics of all participants

		Age	School Grade	BAI total	BDI total	PCL-5 total	STP	STN	PITP	PITN	CITP	CITN	FATP	FATN	CTS-S P	CTS-S N	Which floor
PCL-5 B	**r**	**-0.181** ^**^	**-0.232** ^**^	**0.682** ^**^	**0.609** ^**^	**0.920** ^**^	**-0.135** ^**^	**0.399** ^**^	0.079	**0.406** ^**^	0.003	**0.270** ^**^	0.064	**315** ^**^	-0.045	**0.521** ^**^	0.063
	**p**	**0.000**	**0.000**	**0.000**	**0.000**	**0.000**	**0.002**	**0.000**	0.088	**0.000**	0.946	**0.000**	0.225	**0.000**	0.486	**0.000**	0.126
PCL-5 C	**r**	**-0.154** ^**^	**-0.196** ^**^	**0.573** ^**^	**0.551** ^**^	**0.844** ^**^	-0.75	**0.336** ^**^	0.056	**0.372** ^**^	0.003	**0.215** ^**^	0.053	**0.220** ^**^	-0.016	**0.401** ^**^	0.013
	**p**	**0.000**	**0.000**	**0.000**	**0.000**	**0.000**	0.087	**0.000**	0.231	**0.000**	0.954	**0.000**	0.322	**0.000**	0.808	**0.000**	0.749
PCL-5 D	**r**	**-0.179** ^**^	**-0.235** ^**^	**0.667** ^**^	**0.629** ^**^	**0.915** ^**^	**0.098** ^**^	**0.337** ^**^	0.045	**0.427** ^**^	0.015	**0.270** ^**^	0.066	**0.294** ^**^	.-043	**0.487** ^**^	0.005
	**p**	**0.000**	**0.000**	**0.000**	**0.000**	**0.000**	**0.025**	**0.000**	0.339	**0.000**	0.726	**0.000**	0.216	**0.000**	0.503	**0.000**	0.900
PCL-5 E	**r**	**-0.173** ^**^	**-0.232** ^**^	**0.675** ^**^	**0.633** ^**^	**0.913** ^**^	-0.060	**0.359** ^**^	0.053	0.415	0.015	**0.292** ^**^	0.058	**0.281** ^**^	-0.004	**0.495** ^**^	-0.006
	**p**	**0.000**	**0.000**	**0.000**	**0.000**	**0.000**	0.176	**0.000**	0.258	0.000	0.819	**0.000**	0.275	**0.000**	0.953	**0.000**	0.881
PCL-5 total	**r**	**-0.137** ^**^	-0.229	**734** ^**^	**680** ^**^	1	-0.69	**0.396** ^**^	0.018	**0.389** ^**^	0.012	**0.342** ^**^	0.075	**0.263** ^**^	-0.009	**0.447** ^**^	0.006
	**p**	**0.001**	0.000	**0.000**	**0.000**		0.121	**0.000**	0.707	**0.000**	0.789	**0.000**	0.165	**0.000**	0.892	**0.000**	0.878
BAI total	**r**	**0.562** ^**^	**562** ^**^	1	**0.632** ^**^	**0.734** ^**^	-0.059	**0.369** ^**^	0.020	**0.382** ^**^	0.005	**0.288** ^**^	0.055	**0.231** ^**^	-0.004	**0.376** ^**^	0.017
	**p**	**0.000**	**0.000**		**0.000**	**0.000**	0.189	**0.000**	0.675	**0.000**	0.916	**0.000**	0.305	**0.000**	0.957	**0.000**	0.688
BDI total	**r**	-0.023	**-0.091** ^*^	**0.632** ^**^	1	**0.680** ^**^	-0.016	**0.378** ^**^	0.038	0.491	0.006	**0.300** ^**^	0.054	**0.286** ^**^	0.048	**0.455** ^**^	-0.016
	**p**	0.585	**0.028**	**0.000**		**0.000**	0.716	**0.000**	0.424	**0.000**	0.900	**0.000**	0.312	**0.000**	0.457	**0.000**	0.697

BAI: Beck Anxiety Inventory BDI: Beck Depression Inventory PCL-5: Posttravmatik Stress Disorder Checklist for DSM-5 STP: Survival Traumas positive STN: Survival Traumas negative PITP: Personal Identity Traumas positive PITN: Personal Identity Traumas negative CITP: Collective Identity Traumas positive CITN: Collective Identity Traumas negative FATP: Family-Attachment Traumas positive FATN: Family-Attachment Traumas negative CTS-S P: Cumulative trauma positive CTS-S N: Cumulative trauma positive

*: Correlation is significant at the 0.05 level

**: Correlation is significant at the 0.01 level

Then we evalauted the correlations of PTSD, anxiety and depression scores with CST-S and other clinical characteristics for earthquake exposed group. Correlations were similar to the general participants group. STN, PITN, CITN, FATN scores of CST-S were positively correlated with BAI, BDI and PCL-5 scores although there was no correlation between CST-S positive scores and BAI, BDI and PCL-5 scores.

Correlations of PTSD, anxiety and depression scores with CST-S and other clinical characteristics of all participants are illustrated in Table 3.

**Table 3:** Correlations of PTSD, anxiety and depression scores with CST-S scores and other clinical characteristics of earthquake exposed group

		Age	School Grade	BAI total	BDI total	PCL-5 total	STP	STN	PITP	PITN	CITP	CITN	FATP	FATN	CTS-S P	CTS-S N	Which floor
PCL-5 B	**r**	**-0.169** ^**^	**-0.235** ^**^	**0.709** ^**^	**0.609** ^**^	**0.933** ^**^	**-0.179** ^**^	**0.379** ^**^	0.013	**0.280** ^**^	-0.102	**0.317** ^**^	-0.123	**199** ^*^	-0.038*	**0.280** ^**^	0.0689
	**p**	**0.008**	**0.000**	**0.000**	**0.000**	**0.000**	**0.012**	**0.000**	0.866	**0.000**	0.151	**0.000**	0.129	**0.015**	0.207	**0.006**	0.256
PCL-5 C	**r**	**-0.124**	**-0.205** ^**^	**0.641** ^**^	**0.582** ^**^	**0.889** ^**^	-0.136	**0.333** ^**^	0.016	**0.286** ^**^	0.044	**0.285** ^**^	-0.122	**0.213** ^**^	-0.113	**0.240** ^*^	0.110
	**p**	**0.052**	**0.001**	**0.000**	**0.000**	**0.000**	0.055	**0.000**	0.836	**0.000**	0.536	**0.000**	0.131	**0.009**	0.265	**0.021**	0.624
PCL-5 D	**r**	**-0.163** ^*^	**-0.231** ^**^	**0.730** ^**^	**0.671** ^**^	**0.963** ^**^	**0.168** ^**^	**0.408** ^**^	0.041	**0.346** ^**^	0.111	**0.315** ^**^	-0.096	**0.265** ^**^	.-207	**0.396** ^**^	0.082
	**p**	**0.011**	**0.000**	**0.000**	**0.000**	**0.000**	**0.018**	**0.000**	0.585	**0.000**	0.120	**0.000**	0.236	**0.001**	0.041	**0.000**	0.742
PCL-5 E	**r**	**-0.165** ^*^	**-0.218** ^**^	**0.710** ^**^	**0.656** ^**^	**0.959** ^**^	-0.140	**0.400** ^**^	0.014	0.273**	0.102	**0.310** ^**^	0.113	**0.228** ^**^	-0.150	**0.292** ^**^	-0.073
	**p**	**0.011**	**0.001**	**0.000**	**0.000**	**0.000**	0.050	**0.000**	0.850	**0.000**	0.153	**0.000**	0.164	**0.005**	0.141	**0.005**	0.537
PCL-5 total	**r**	**-0.174** ^**^	-0.224**	**745** ^**^	**668** ^**^	1	-0.167*	**0.404** ^**^	0.009	**0.330** ^**^	-0.099	**0.327** ^**^	-0.104	**0.227** ^**^	-0.186	**0.340** ^**^	0.001
	**p**	**0.008**	**0.001**	**0.000**	**0.000**		0.021	**0.000**	0.904	**0.000**	0.170	**0.000**	0.210	**0.006**	0.071	**0.001**	0.639
BAI total	**r**	**-0.121**	**144** ^*^	1	**0.605** ^**^	**0.745** ^**^	-0.164*	**0.351** ^**^	0.013	**-0.155** ^*^	0.005	**0.244** ^**^	-0.095	**0.210** ^*^	-0.217*	**0.264** ^*^	0.009
	**p**	**0.067**	**0.029**		**0.000**	**0.000**	0.025	**0.000**	0.871	**0.034**	0.155	**0.001**	0.254	**0.012**	0.035	**0.012**	0.988
BDI total	**r**	-0.064	**-0.091**	**0.635** ^**^	1	**0.668** ^**^	-0.084	**0.403** ^**^	0.044	0.456**	0.103	**0.242** ^**^	-0.016	**0.255** ^**^	0.063	**0.306** ^**^	-0.112
	**p**	0.331	**0.164**	**0.000**		**0.000**	0.247	**0.000**	0.563	**0.000**	0.150	**0.001**	0.843	**0.002**	0.537	**0.003**	0.761

BAI: Beck Anxiety Inventory BDI: Beck Depression Inventory PCL-5: Posttravmatik Stress Disorder Checklist for DSM-5 STP: Survival Traumas positive STN: Survival Traumas negative PITP: Personal Identity Traumas positive PITN: Personal Identity Traumas negative CITP: Collective Identity Traumas positive CITN: Collective Identity Traumas negative FATP: Family-Attachment Traumas positive FATN: Family-Attachment Traumas negative CTS-S P: Cumulative trauma positive CTS-S N: Cumulative trauma positive

*: Correlation is significant at the 0.05 level

**: Correlation is significant at the 0.01 level

### Evaluation of predictors for PTSD, anxiety and depression

Logistic regression analysis was performed to evaluate the predictors for PTSD, anxiety, depression in all participants and in earthquake exposed group. Results of logistic regression analysis for all participants are illustrated in Table 4 and results of logistic regression analysis for all earthquake exposed group are illustrated in Table 5.

**Table 4:** Logistic regression analysis for PTSD, anxiety and depression

Model	Unstandardized Coefficients		Standardized Coefficients	t	Sig.	Correlations			Collinearity Statistics	
	B	Std.Error	Beta			Zero-order	partial	part	tolerence	VIF
**Dependent variable PCL-5 total**										
1 (Constant)	43.514	8.807	-0.055	4.941	**0.000**					
CTS-S total positive	-0.122	0.084		-1.459	0.146	-0.023	-0.103	-0.054	0.975	1.026
Age	-1.799	0.385	-0.182	-4.673	**0.000**	-0.295	-0.314	-0.174	0.920	1.087
STN	0.241	0.111	0.097	2.181	**0.030**	0.399	0.152	0.081	0.700	1.429
PITN	-0.107	0.089	-0.061	-1.198	.**032**	0.414	-0.084	-0.045	0.530	1.887
CTN	0.216	0.092	0.103	2.355	**0.019**	0.279	0.164	0.088	0.726	1.378
FATN	0.073	0.135	0.024	0.545	0.586	0.256	0.039	0.020	0.712	1.405
BDI total	0.860	0.112	0.373	7.659	**0.000**	0.665	0.476	0.286	0.588	1.701
BAI total	0.800	0.075	0.489	10.593	**0.000**	0.757	0.600	0.396	0.654	1.528
**Dependent variable anxiety**										
1 (Constant)	-1.372	6.993		-0.196	0.845					
CTS-S total positive	0.046	0.063	0.034	0.731	0.466	0.020	0.052	0.033	0.967	1.034
Age	0.097	0.304	0.016	0.318	0.751	-0.205	0.022	0.015	0.830	1.205
PCL-5 total	0.449	0.042	0.735	10.593	**0.000**	0.757	0.600	0.485	0.435	2.298
STN	0.069	0.084	0.046	0.829	0.408	0.343	0.059	0.038	0.686	1.458
PITN	0.094	0.067	0.089	1.412	0.159	0.376	0.099	0.065	0.531	1.882
CTN	-0.039	0.070	-0.031	-0.562	0.575	0.221	-0.040	-0.026	0.707	1.414
FATN	-0.068	0.101	-0.037	-0.679	0.498	0.201	-0.048	-0.031	0.713	1.403
BDI total	-0.021	0.096	-0.015	-0.224	0.823	0.519	-0.016	-0.010	0.455	2.199
**Dependent variable depression**										
1 (Constant)	-7.038	5.139		-1.369	0.172					
CTS-S total positive	0.021	0.047	0.022	0.450	**0.653**	0.035	0.032	0.021	0.965	1.036
Age	0.489	0.222	0.114	2.207	**0.028**	-0.078	0.154	0.105	0.849	1.177
PCL-5 total	0.264	0.034	0.608	7.659	**0.000**	0.665	0.476	0.365	0.361	2.773
BAI total	-0.012	0.052	-0.017	-0.224	0.823	0.519	-0.016	-0.011	0.419	2.385
STN	-0.034	0.062	-0.032	-0.551	0.582	0.304	-0.039	-0.026	0.685	1.461
PITN	0.272	0.046	0.360	5.941	**0.000**	0.541	0.387	0.283	0.619	1.616
CTN	-0.095	0.051	-0.104	-1.857	0.065	0.228	-0.130	-0.089	0.718	1.392
FATN	-0.020	0.075	-0.015	-0.263	0.793	0.252	-0.019	-0.013	0.711	1.406

BAI: Beck Anxiety Inventory BDI: Beck Depression Inventory PCL: Posttravmatik Stress Disorder Checklist for DSM-5 STP: Survival Traumas positive STN: Survival Traumas negative PITP: Personal Identity Traumas positive PITN: Personal Identity Traumas negative CITP: Collective Identity Traumas positive CITN: Collective Identity Traumas negative FATP: Family-Attachment Traumas positive FATN: Family-Attachment Traumas negative CTS-S P: Cumulative trauma positive CTS-S N: Cumulative trauma positive

**Table 5:** Logistic regression analysis for PTSD, anxiety and depression of earthquake exposed group

Model	Unstandardized Coefficients		Standardized Coefficients	t	Sig.	Correlations			Collinearity Statistics	
	B	Std.Error	Beta			Zero-order	partial	part	tolerence	VIF
**Dependent variable PCL-5 total**										
1 (Constant)	70.373	24.882	-0.022	2.828	**0.006**					
CST-S total positive	-0.047	0.155		-0.304	0.762	-0.209	-0.037	-0.021	0.884	1.131
Age	-3.013	1.325	-0.256	-2.275	**0.026**	-0.218	-0.268	-0.158	0.382	2.619
STN	0.361	0.179	0.186	2.014	**0.048**	0.384	0.239	0.140	0.567	1.763
PITN	-0.189	0.153	-0.115	-1.239	0.220	0.249	-0.150	-0.086	0.557	1.796
CTN	0.174	0.150	0.092	1.158	0.251	0.185	0.140	0.080	0.767	1.304
FATN	0.078	0.209	0.031	0.374	0.709	0.154	0.046	0.026	0.689	1.452
BDI total	0.817	0.183	0.385	4.459	**0.000**	0.585	0.478	0.310	0.647	1.546
BAI total	0.783	0.142	0.473	5.506	**0.000**	0.718	0.558	0.383	0.654	1.529
School Grade	0.207	1.587	0.014	0.131	0.896	-0.205	0.016	0.009	0.396	2.524
Psychosocial Support	-11.01	6.903	-0.118	-1.595	0.115	0.090	-0.191	-0.111	0.882	1.134
**Dependent variable anxiety**										
1 (Constant)	-14.15	18.693		-0.757	0.452					
CST-Stotal positive	-0.097	0.110	-0.077	-0.885	0.379	-0.216	-0.108	-0.073	0.893	1.120
Age	0.878	0.974	0.123	0.901	0.371	-0.102	0.109	0.074	0.359	2.788
PCL-5 total	0.398	0.072	0.658	5.506	**0.000**	0.718	0.558	0.451	0.470	2.128
STN	0.086	0.131	0.073	0.655	0.515	0.365	0.080	0.054	0.538	1.858
PITN	0.066	0.110	0.067	0.600	0.550	0.254	0.073	0.049	0.547	1.828
CTN	-0.029	0.108	-0.025	-0.266	0.791	0.136	-0.032	-0.022	0.753	1.329
FATN	-0.057	0.149	-0.038	-0.381	0.705	0.159	-0.046	-0.031	0.689	1.452
BDI total	0.008	0.149	0.006	0.056	0.956	0.471	0.007	0.005	0.499	2.005
School Grade	-0.909	1.127	-0.105	-0.807	0.423	-0.132	-0.098	-0.066	0.400	2.500
Psychosocial Support	6.686	4.948	0.119	1.351	0.181	0.187	0.163	0.111	0.873	1.145
**Dependent variable depression**										
1 (Constant)	-25.61	15.093		-1.697	0.094					
CST-S total positive	-0.037	0.091	-0.037	-0.404	0.688	-0.124	-0.049	-0.035	0.885	1.130
Age	1.508	0.784	0.272	1.925	0.059	0.048	0.229	0.166	0.374	2.674
PCL-5 total	0.280	0.063	0.594	4.459	**0.000**	0.585	0.478	0.385	0.420	2.383
BAI total	0.006	0.100	0.007	0.056	0.956	0.471	0.007	0.005	0.450	2.221
STN	-0.157	0.106	-0.171	-1.476	0.145	0.252	-0.177	-0.127	0.552	1.811
PITN	0.280	0.084	0.362	3.348	**0.001**	0.419	0.379	0.289	0.635	1.574
CTN	-0.082	0.088	-0.092	-0.931	0.355	0.094	-0.113	-0.080	0.762	1.313
FATN	-0.053	0.123	-0.045	-0.432	0.667	0.191	-0.053	-0.037	0.689	1.451
School Grade	-0.686	0.926	-0.101	-0.741	0.461	-0.002	-0.090	-0.064	0.399	2.504
Psychosocial Support	8.021	4.000	0.182	2.006	**0.049**	0.225	0.238	0.173	0.901	1.110

BAI: Beck Anxiety Inventory BDI: Beck Depression Inventory PCL-5: Posttravmatik Stress Disorder Checklist for DSM-5 STP: Survival Traumas positive STN: Survival Traumas negative PITP: Personal Identity Traumas positive PITN: Personal Identity Traumas negative CITP: Collective Identity Traumas positive CITN: Collective Identity Traumas negative FATP: Family-Attachment Traumas positive FATN: Family-Attachment Traumas negative CTS-S P: Cumulative trauma positive CTS-S N: Cumulative trauma positive

## Discussion

In this study, we examined the impact of the 2023 Great Anatolian earthquake on the prevalence of PTSD, depression, and anxiety, and researched the question as to whether socio-demographic, earthquake exposure-related and clinical factors such as cumulative trauma are associated with the presence of these diagnoses. To our knowledge, this is the first study conducted in our country regarding this earthquake. Although there is research on earthquakes and their impact on PTSD, depression, and anxiety, there are not many studies evaluating the relationship between emerging mental disorders and cumulative trauma.

The prevalence rates of PTSD, anxiety, and depression were 38.9%, 28.7%, and 21.1%, respectively. In terms of PTSD, findings were consistent with previous studies. A systematic review by Neria et al. [22].reported that prevalence of PTSD among victims of a disaster generally ranges from 30 to 40%. There are also studies that found a much lower prevalence of 23.4% four years after an earthquake [23].or only 13.10% three years after an earthquake [24].Since our study was carried out two months after earthquake, it can be expected that rates are higher than those of these studies. As a result, it may be possible for symptom severity of PTSD to improve over time [25].

There are reports reporting PTSD rate of between 50 and 68% among children exposed to earthquakes [26] and a study evaluating adolescents after hurricane also reported PTSD at a rate of 57.5% [27]. The older mean age of the participants in our study may be a reason for lower rate.

In our study, rate of depression was found to be 21.1%. In the study of Cenat et al. [28] who conducted a systematic review and meta-analysis evaluating PTSD, depression, and anxiety after earthquake, median prevalence of depression was reported as 32.16% and GA 95% (23.60-42.11%), obtained from the meta-analysis of 14 studies reporting depression, but the results showed heterogeneity according to age and post-earthquake duration, and depression rates were reported lower in adults. Although it was reported that prevalence decreased over time compared to time after the earthquake [25], it can be said that the rate was low even two months after earthquake. Placement of university students in a university and dormitories in another city shortly after earthquake and their continuing education may be protective against depression. One study reported that children attending school were at higher risk of experiencing greater symptoms of PTSD compared to children who did not after earthquake [29]. However, in that study, there were children continue living in same region. Being transferred to a different province or continuing their education may also have helped to stay away from the traumatic effects of situations such as earthquake debris, prefabricated lives, which may be reminder factors.

The anxiety rate in our study was 28.7%. Data on the prevalence of anxiety after earthquake are scarce. One study found the prevalence of anxiety symptoms 28 months after earthquake, 21% [30]. The period and age after earthquake may be effective in these results.

The discrepancy between studies can be explained by various factors such as different tools used, time after exposure, severity of disaster, previous traumatic experience, and other variables [30].

Women showed a higher rate for all three diagnoses. PTSD, depression and anxiety symptoms were negatively correlated with age and school grade. The fact that prevalence rates are lower than those of children and adolescents and this negative correlation with age may suggest that age is protective. These data are in line with authors who suggested that women and younger individuals are more vulnerable to traumatic events and more prone to develop post-traumatic stress symptoms when exposed to trauma [30, 31].

Residing in earthquake province and being there during earthquake was higher for all three diagnoses. The rate of witnessing injury, death, participating in search-rescue efforts, and having a close intimate under dent, was high for three diagnoses. The rate of mild-moderate-heavy damaged hauses was higher in all three. This may suggest that uncertainty about risk of collapse increases anxiety [32]. Previous studies conducted following earthquake showed that the level of exposure to traumatic event was one of the most important predictor of PTSD, depression, and anxiety symptoms, both for children, adolescents [24], and adults [25]. For instance, those who experienced death/injury of a family member were at increased risk of PTSD, depression and anxiety [32, 33]. One of the influencing factors, having a previous psychiatric history in self/family, was common in PTSD, anxiety and depression. Our result consistent with the study of Cadichon et al. [34] who reported that adolescents and young adults with parents with previous psychological problems have an increased risk of PTSD. Similarly, studies conducted on impacts of indirect exposure to earthquake found that being born in earthquake district and having friends and family living in earthquake district during the event was related to these symptoms [35, 36].

Another evaluation that could be impressive for all three diagnoses was cumulative trauma. In scoring of cumulative trauma, negative evaluation scores were associated with all three diagnoses.

STN, PITN, CITN, FATN scores were positively correlated with PTSD, anxiety, and depression. Various studies have estimated that severity of psychiatric symptoms are more severely in individuals exposed to multiple traumas than a single event [19, 37]. Experiencing a natural disaster, being physically attacked, witnessing a violent attack, war, terrorist attack are the most common survival traumas. As survival trauma increases, psychiatric symptoms also increase [37].

PIT negatively affect people’s self-esteem and can cause people to feel that they do not belong to any environment. It includes sexual traumas and relational traumas [21] Personal identity trauma has negative effects on cognitive functions such as procedural memory and executive functions [20]. Sexual abuse is one of the traumas that cause most emotional harm, especially in childhood [38]. Studies report personality trauma as an important predictor of psychiatric disorders [20]. Financial difficulties are also stated as triggers of various psychiatric disorders, including anxiety, mood and behavioral disorders [38].

CIT occurs when the person is marginalized by others depending on factors such as religion, language, race, etc [38]. Kira et al. [19, 20] found in their studies that CITs are traumas that most strongly affect mental health and it was the most damaging trauma types.

FATs are especially childhood traumas and one of the most important determinants for PTSD [19].

Traumatic situations can cause people to experience mental disorders [39]. When a traumatic experience is experienced, as a result of a higher level of stimuli than the self can cope with, psychiatric disorders such as PTSD, anxiety, depression, ocur [40, 41]. State that the meaning that a person attributes to the traumatic situation and threat of death he perceives at the time of the traumatic situation are determinants of depression, anxiety and PTSD symptoms.

According to Kira et al. accumulation and dynamics of different types of trauma are affected by cumulative positive or negative evaluations and play a role in coping with stress. In addition, when evaluations are negative, they contribute to maladaptive coping, and when they are positive, they contribute to active coping and recovery [20]. In our results, especially the predictive effect of negative evaluation scores on clinical symptoms supports these results.

PTSD, depression, and anxiety were significantly associated with earthquake exposure as well as cumulative trauma. The findings have important implications for disaster preparedness, mental and psychological interventions, and mental health prevention. Special mental and psychological interventions should be provided to those who have or are at risk of developing mental disorders, especially those with high negative scores of cumulative trauma. Psychological first aid can be considered as one of the early interventions for disaster victims. Health facilities should be improved to have a strengthened mental health service. In universities, mental health services should be part of the routine health care system to assist with mental health prevention goals. Additionally, future studies should consider several variables, such as the impact of local wisdom on disaster preparedness, post-disaster social support, local culture, and mental health-seeking behaviors.

### Limitations

Despite our significant findings, the study also had some limitations. Because it is a cross-sectional design, it doesn’t provide information about changes in mental health problems over time. Second, although the instruments used in this study had satisfactory psychometric properties, they were considered as a screening measure to identify potential clinical cases of mental disorders. Using a standard clinical interview will be important to assess the prevalence of PTSD, depression, and anxiety among students in this region.

## Conclusion

Negative evaluation scores of cumulative trauma are predictive on PTSD, depression and anxiety, in addition to gender, previous psychiatric diagnosis and high scores of earthquake-related features. But it may be more meaningful to support these results with longitudinal studies with clinical evaluation.

## Data Availability

No datasets were generated or analysed during the current study.
